# Myo-inositol improves the host’s ability to eliminate balofloxacin-resistant *Escherichia coli*

**DOI:** 10.1038/srep10720

**Published:** 2015-06-01

**Authors:** Xin-hai Chen, Bing-wen Zhang, Hui Li, Xuan-xian Peng

**Affiliations:** 1 Center for Proteomics and Metabolomics, State Key Laboratory of Bio-Control, MOE Key Lab Aquat Food Safety, School of Life Sciences, Sun Yat-sen University, University City, Guangzhou 510006, People’s Republic of China; 2 Drug Discovery Pipeline, Guangzhou Institutes of Biomedicine and Health, Chinese Academy of Sciences, Guangzhou 510530, Guangdong, People’s Republic of China

## Abstract

Antibiotic-resistant mechanisms are associated with fitness costs. However, why antibiotic-resistant bacteria usually show increasing adaptation to hosts is largely unknown, especially from the host’s perspective. The present study reveals the host’s varied response to balofloxacin-resistant *Escherichia coli* (BLFX-R) using an integrated proteome and metabolome approach and identifies myo-inositol and phagocytosis-related proteins as crucial biomarkers. Originally, macrophages have an optimal attractive preference to BLFX-S due to more polarization of BLFX-S than BLFX-R, which renders faster elimination to BLFX-S than BLFX-R. The slower elimination to BLFX-R may be reversed by exogenous myo-inositol. Primarily, myo-inositol depolarizes macrophages, elevating adherence to both BLFX-S and BLFX-R. Since the altered adherence is equal to both strains, the myo-inositol-treated macrophages are free of the barrier to BLFX-R and thereby promote phagocytosis of BLFX-R. This work provides a novel strategy based on metabolic modulation for eliminating antibiotic-resistant bacteria with a high degree of host adaptation.

Antimicrobial resistance in bacterial pathogens has become a serious threat to public health, animal feed and aquaculture, despite our efforts to halt the increase and spread of this resistance^1^,^2^. One reason for this is the speed with which bacteria develop resistance to antibiotics is in contrast to the slow development of new drugs^2^,^3^. Furthermore, the antibiotic pipeline is in a drought^2^,^3^,^4^. Thus, it is essential to explore new paradigms for anti-infective therapy as an alternative strategy against antibiotic resistance, and host-directed immune modulatory therapies may be one promising approach^5^.

A line of evidence has indicated that antimicrobial resistance could be associated with fitness cost in bacteria. There is a general notion that the acquisition of drug resistance, particularly the resistance mediated by chromosomal mutations, entails a biological cost for pathogens, resulting in reduced fitness in the absence of antibiotic selection pressure, such as a lower growth rate and bacteria that are initially less invasive or transmissible than their susceptible counterparts^1^,^2^. However, *in vivo* selected or clinically derived isolates may develop compensatory mutations that reduce the fitness cost associated with antimicrobial resistance^3^,^4^,^5^,^6^,^7^. Different environments (*in vitro* vs. *in vivo*) may select for different compensatory mutations and varied levels of restoration of fitness^8^,^9^. One important finding is that clinically derived drug-resistant isolates display diverse fitness changes^4^,^10^, generally showing adaptation to host environments and thereby exhibiting longer survival in the host. This may be the reason why drug-resistant bacteria are not easily eliminated by the synergistic effects of host immunity and antibiotics, consequently leading to chronic infections^11^,^12^. Seemingly, the costs of having a lower growth rate and being less invasive or initially transmissible are compensated for by adaptation to hosts, which contributes to a balance between the hosts and the antibiotic-resistant pathogens. In this regard, longer survival in the host can be attributed to both the pathogens and the hosts. The coordination between the hosts and pathogens determines the enhancement of adaptations of bacterial pathogens to hosts. However, our understanding of fitness is generally obtained from a bacterial perspective^1^,^2^. Information regarding to the adaptation permitted by the hosts is largely unknown.

With regard to the general mechanism of the permission allowed by hosts, a global regulation that decelerates the elimination of antibiotic-resistant pathogens from the hosts may often play a role. Although this regulation is incompletely understood, it seems likely that it includes alterations of protein expression and metabolic regulation, some of which could be modulated by the extracellular environment^13^,^14^. This hypothesis is investigated in the balofloxacin-resistant *Escherichia coli* (BLFX-R) experiments described below.

## Results and Discussion

### Balofloxacin-resistant *E. coli* show high adaptation *in vivo*

**Figure 1:**
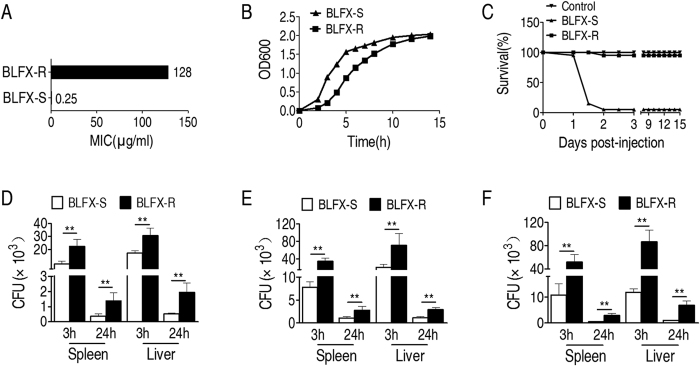
Fitness of BLFX-R *in vitro* and *in vivo*. (**A**) MIC of BLFX-R and BLFX-S. (**B**) Growth curves of BLFX-R and BLFX-S. (**C**) Survival curve of zebrafish infected with BLFX-R or BLFX-S. (**D**–**F**) Elimination of BLFX-R in a mouse model. Mixtures with three ratios of BLFX-S and BLFX-R were injected into mice. The three ratios of BLFX-S and BLFX-R consisted of 1.14:1 (**D**), 1:1.75 (**E**) and 1:3.5 (**F**). Spleens and livers were obtained at 3 h and 24 h after injection and homogenized in an ice bath. Bacterial cells were determined via viable plate-counting. Error bars ± s .e .m, ^*^ *p* < 0.05, ^**^ *p* < 0.01.

To obtain BLFX-R, *E. coli* K12 BW25113 was subcultured in LB medium with and without a 1/2 MIC of balofloxacin for serial passages, which led to a 512-fold increased and unchanged MIC, resulting in BLFX-R and control balofloxacin-sensitive *E. coli* (BLFX-S), respectively (Fig. 1A). We tested the fitness of the two strains *in vitro* by measurement of growth in LB medium and found significantly less growth of BLFX-R than BLFX-S before the stationary phase was reached (Fig. 1B). This is consistent with previous observation that antibiotic resistance is associated with fitness costs^15^,^16^. Then, we investigated the difference in pathogenicity between BLFX-R and BLFX-S in a zebrafish model. BLFX-S caused serious mortality of zebrafish, while BLFX-R showed weak pathogenicity (Fig. 1C), suggesting an enhanced adaptation of BLFX-R in zebrafish. We further tested whether the enhanced adaptation might result in longer survival of BLFX-R than BLFX-S in a mouse model. The two strains at three different ratios comprising a total dose of 5 × 10^7^ CFU were intraperitoneally injected into each mouse. More BLFX-R than BLFX-S cells were detected in the spleens and livers of the mice treated with all three of the ratios of the two bacteria at 3 and 24 h after infection (Fig. 1D–1F). Taken together, these data indicate that BLFX-R presented higher host compatibility than BLFX-S and was hardly eliminated by hosts.

### Integrated proteomic and metabolomic analysis identifies myo-inositol and phagocytosis-related proteins as crucial biomarkers

**Figure 2:**
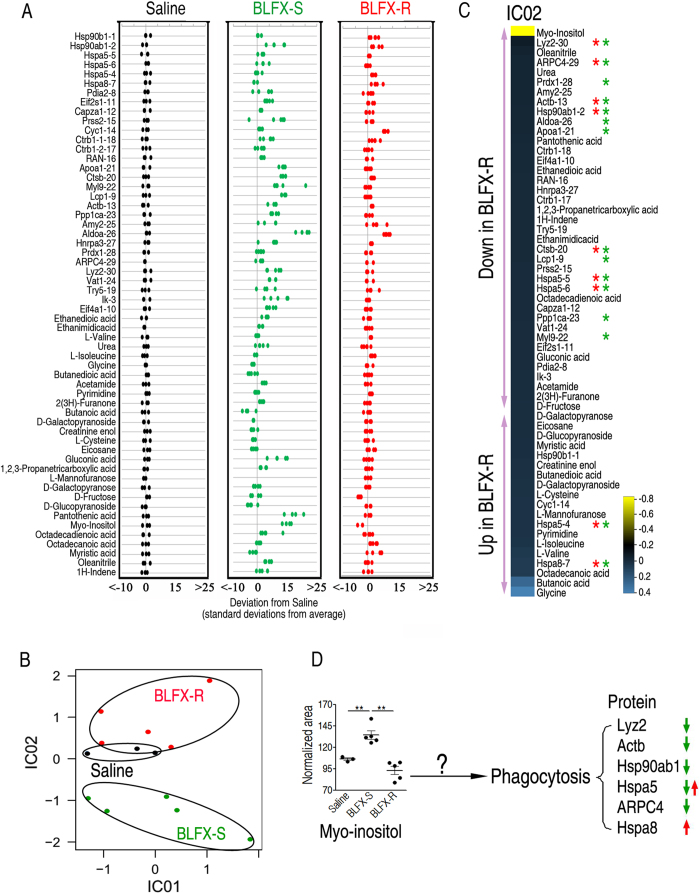
Bioinformatic analysis of the integration of metabolomic and proteomic profiling. (**A**) *Z*-score plots for the integrated protein-metabolite data normalized to the mean of the saline-injected mouse spleen samples. (black, sterile saline; green, BLFX-S; red, BLFX-R). (**B**) ICA analysis of integrated protein and metabolite profiles from the spleen in response to BFLX-S or BFLX-R. (**C**) IC02 of the differential abundance of proteins and metabolites. Ranked loadings for the corresponding IC02 were selected for representation. Positive and negative values indicate increased and decreased proteins or metabolites in BLFX-S compared to BLFX-R. Red and green asterisks indicate the proteins related to immunity and phagocytosis, respectively. Protein spot names and numbers are indicated (such as Gapdh-8307). (**D**) Hypothesizing the existence of a potential link between myo-inositol and phagocytosis. Error bars ± s .e .m, ^*^ *p* < 0.05, ^**^ *p* < 0.01.

The spleen plays important roles in anti-infective immunity. For this reason, we selected the spleen as a targeted organ and explored the global alteration by which the host permits BLFX-R to survive longer using an integrated proteomics and metabolomics approach. Five (injection of BLFX-S or BLFX-R) and three (injection of sterile saline) biological repeats for proteomics or metabolomics analysis, and three and two technical replicates for proteomics and metabolomics analysis, respectively, were performed in each group, thereby yielding a total of 65 samples. Approximately 910 protein spots were detected on 2-DE gels. Out of the 910 protein spots, 30 spots representing 27 proteins showed significant differences in abundance from BLFX-R compared with BLFX-S, which were identified using MALDI-TOF/MS (see Supplementary Table S1 online) and are shown in Supplementary Fig. S1 online. In the metabolomics analysis, seventy-five metabolites were detected in each sample (see Supplementary Fig. S2 online). Of these metabolites, twenty–six differential abundances of metabolites were identified in BLFX-R compared to BLFX-S (see Supplementary Fig. S2 online). We integrated these differential proteomics and metabolomics data with a combined covariance/correlation matrix of proteins and metabolites as described previously^17^. The combined proteins and metabolites are displayed as a *z*-score plot, in which saline-based *z*-scores are plotted for each protein or metabolite (Fig. 2A). ICA, based on the integrated data, was used to differentiate BLFX-R from BLFX-S by IC02 (Fig. 2B). Of the IC02 loadings, 39 were decreased and 19 were increased in BLFX-R (Fig. 2C). Myo-inositol showed the largest impact (Fig. 2C) with decreased and increased abundance in mice infected with BLFX-R and BLFX-S, respectively (Fig. 2D). The biological pathway analysis for these varied proteins (software is not available for the integrated analysis of both proteins and metabolites) indicated that 13 proteins contribute to host immunity in which six proteins participate in phagocytosis, including Lyz2, Actb, Hsp90ab1, Hspa5, ARPC4 and Hspa8. The proteins involved in immunity and phagocytosis were indicated by red and green asterisks, respectively (Fig. 2C). Except for Hspa8 and Hspa5, all proteins exhibited a decrease in BLFX-R, indicating ineffective phagocytosis in the host in response to drug-resistant bacteria. Given that, a becoming possibility is that ineffective phagocytosis may be related to a decreased abundance of myo-inositol because this metabolite showed the highest impact in mice infected by BLFX-R (Fig. 2C,D). It is therefore rational to examine whether exogenous myo-inositol may restore host’s ability to eliminate drug-resistant bacteria.

### Exogenous myo-inositol induces the host to eliminate BLFX-R

**Figure 3:**
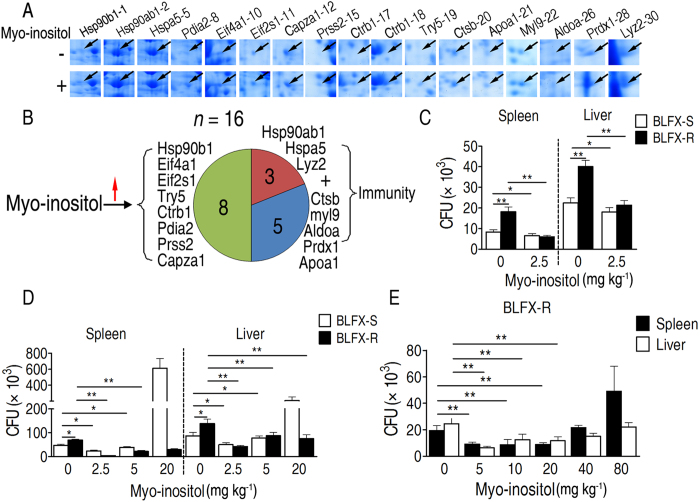
Exogenous myo-inositol promotes host elimination of BFLX-R *in vivo*. (**A**) Enlarged map of 17 protein spots representing 16 protein entries increased in abundance in the presence of exogenous myo-inositol. (**B**) Distribution of the 16 proteins from (**A**). Eight proteins contribute to host immunity, among which three proteins function in phagocytosis. (**C**) The bacterial load of spleens and livers of mice that were injected with or without myo-inositol and then challenged by a mixture consisting of equal amounts of BLFX-R and BLFX-S. (**D**) The bacterial load of spleens and livers of mice that were injected with or without myo-inositol and then challenged by equal amounts of BLFX-R or BLFX-S. (**E**) The effect of increasing concentrations of myo-inositol on BFLX-R loading in spleens and livers of mice. Error bars ± s .e .m, ^*^ *p* < 0.05, ^**^ *p* < 0.01.

We first investigated whether exogenous myo-inositol mediates the expression of the above-mentioned twenty-seven varied proteins. Consistently, sixteen proteins were significantly increased in abundance in the myo-inositol-injected mouse spleen (Fig. 3A). Of these proteins, eight are related to immunity and three of these contribute to phagocytosis (Fig. 3B). This finding provides evidence that exogenous myo-inositol has the ability to enhance host immunity, especially phagocytosis, and eradicate the pathogen (Fig. 3B). To further demonstrate this finding, we investigated the functional effect of exogenous myo-inositol on phagocytosis *in vivo*. After treating mice with and without 2.5 mM myo-inositol twice a day for three days, we detected bacterial loads 12 h after injection in the spleen and liver of mice that were injected with a mixture of equal doses of BLFX-R and BLFX-S. Interestingly, the exogenous myo-inositol-injected mice showed a specific restoration of the ability to eliminate BLFX-R besides promoting elimination of BLFX-S (Fig. 3C). This could be further confirmed by the discovery that approximately equal numbers of BLFX-S and BLFX-R cells were detected in 2.5 and 5 mM myo-inositol-injected mice infected respectively with BLFX-R and BLFX-S (Fig. 3D). However, 20 mM myo-inositol-injected mice showed a reduced ability to eliminate BLFX-S (Fig. 3D), suggesting that bacterial elimination ability is decreased with high doses of myo-inositol. This led us to further investigate whether the dose of exogenous myo-inositol was related to the elimination of BLFX-R. We found that restoration of the ability to eliminate BLFX-R did not occur with a high dose of myo-inositol (Fig. 3E). Collectively, these results demonstrate that exogenous low and intermediate doses of myo-inositol may reduce host compatibility of BLFX-R, and ultimately to increase elimination of BLFX-R in mice, providing a complementary or alternative therapeutic intervention for infections caused by antibiotic-resistant bacteria.

### Myo-inositol enables depolarization of macrophages, which attracts BLFX-R

**Figure 4:**
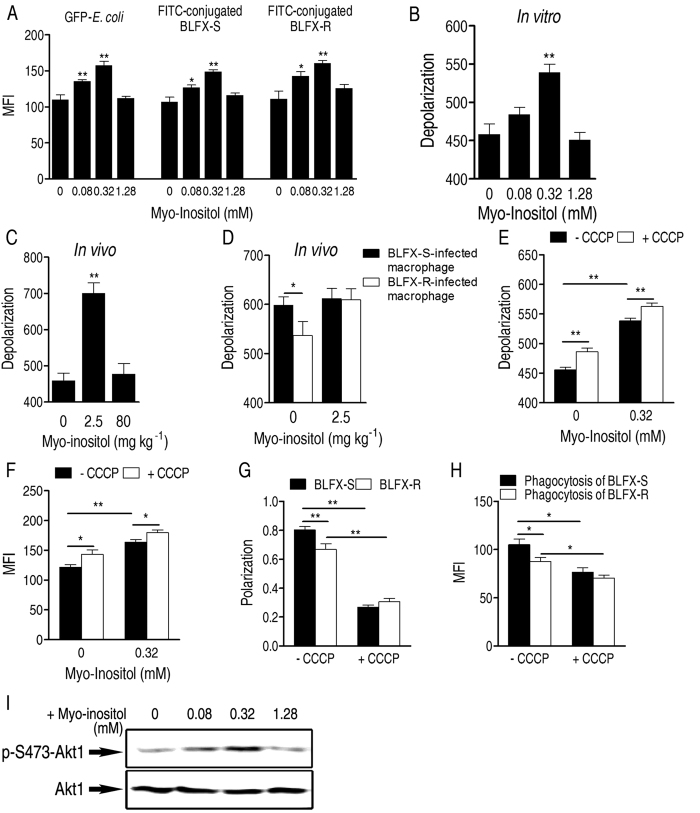
Myo-inositol enhances macrophage-mediated phagocytosis by depolarizing the macrophage membrane potential. (**A**) Mean fluorescence intensity (MFI) showing phagocytosis of GFP-*E. coli,* FITC-conjugated BLFX-S and BLFX-R by myo-inositol-pretreated RAW264.7 cells at the indicated concentrations. (**B**) Depolarization of macrophages in the presence of the indicated concentrations of myo-inositol *in vitro*. (**C**) Effect of pretreatment with myo-inositol on depolarization of macrophages in mice. (**D**) Effect of BLFX-S or BLFX-R on depolarization of macrophages in the presence or absence of myo-inositol. (**E**) Depolarization of macrophages from mice pretreated with or without myo-inositol or/and in the presence or absence of CCCP. (**F**) MFI showing phagocytosis of GFP-*E. coli* by RAW264.7 cells pretreated with CCCP, myo-inositol or a combination of myo-inositol and CCCP. (**G**) Detection of membrane potential in BLFX-S and BLFX-R cells treated with DiOC2(3) (3,3’-Diethyloxacarbocyanine iodide), a membrane potential probe, in the presence or absence of CCCP. (**H**) MFI showing phagocytosis of FITC-conjugated BLFX-S or BLFX-R pretreated with or without CCCP in RAW264.7 cells. (**I**) Phosphorylation of Akt1 in exogenous myo-inositol-stimulated macrophages. Error bars ± s .e .m, ^*^ *p* < 0.05, ^**^ *p* < 0.01.

We hypothesized that the ability to eliminate BLFX-R might be mediated by macrophages because they function in non-specific immune defenses including phagocytosis^18^. To examine this idea, we used flow cytometry to detect the phagocytosis efficiency of RAW264.7, a murine macrophage cell line, with or without pretreatment of myo-inositol. First, we demonstrated that exogenous myo-inositol markedly enhanced phagocytosis in GFP-expressing *E. coli* (Fig. 4A). Next, myo-inositol stimulation also enhanced the phagocytosis of FITC-conjugated BLFX-S and BLFX-R (Fig. 4A). These increases disappeared when a high dose of the metabolite was used (Fig. 4A), which was consistent with the finding that the increased bacterial elimination ability shown *in vivo* disappeared when a high dose of myo-inositol was administered (Fig. 3D). The above results give strong indication that myo-inositol increases macrophage-mediated phagocytosis, which contributes to the enhanced ability to eliminate BLFX-R.

Reports have indicated that myo-inositol was likely involved in phagocytosis^19^,^20^. Given that depolarization contributes to phagocytosis^21^, we reasoned that myo-inositol alters the depolarization of macrophages, and in turn promotes phagocytosis to cope with BLFX-S and BLFX-R. To examine this idea, we first tested whether myo-inositol depolarized macrophages, the depolarization elevated the membrane potential difference between macrophages and bacteria, and thereby increased phagocytosis. Consistent with this hypothesis, low and intermediate doses of exogenous myo-inositol significantly depolarized the membrane potential of macrophages *in vitro* (Fig. 4B). Increased depolarization of macrophage membrane potential was detected *in vivo* when mice were pre-treated with 0.25 mM, but not 80 mM, myo-inositol (Fig. 4C). BLFX-S-infected mice showed more depolarization of the macrophage membrane potential than BLFX-R-infected mice, and this depolarized difference was disappeared when mice were pre-treated with myo-inositol (Fig. 4D). Macrophages were also depolarized by pretreatment with carbonyl cyanide m-chlorophenyl hydrazone (CCCP), a depolarizing reagent, myo-inositol or a combination of myo-inositol and CCCP (Fig. 4E), which contributed to the significantly increased phagocytosis (Fig. 4F). Then, we demonstrated lower polarization of BLFX-R than BLFX-S, which disappeared when mice were pretreated with CCCP (Fig. 4G). Consistently, reduced phagocytosis was detected in BLFX-R compared to BLFX-S infected mice, but a similar amount of phagocytosis was found for mice infected respectively with CCCP-pretreated BLFX-R and BLFX-S (Fig. 4H), indicating that BLFX-R exhibits a less polarized membrane potential, which leads to reduced elimination by the host. Together, these data reveal that the attenuated phagocytosis for BLFX-R, and phagocytosis increased by myo-inositol-pretreated macrophages can be attributed to changes in depolarization.

There are two by-products, phosphatidylinositol-3,4,5-trisphosphate (PtdIns(3,4,5)P3) and inositol 1,3,4,5-tetrakisphosphate (Ins(1,3,4,5)P4) in inositol phosphate metabolism of myo-inositol. PtdIns(3,4,5)P3 binds PH domain-containing proteins, such as Akt1, and triggers downstream signaling pathways leading to various cellular events including polarized alteration^19^,^20^,^22^, whereas Ins(1,3,4,5)P4 functions in suppression of PH domain-containing proteins^20^. We supposed that different concentrations of exogenous myo-inositol led to different ratios between PtdIns(3,4,5)P3 and Ins(1,3,4,5)P4, differentially regulated Akt1 and thereby differentially depolarized the membrane potential of macrophages. Consistently, we found that exogenous low and intermediate doses of myo-inositol stimulate phosphorylation of Akt1 at Ser 473 and high dose of myo-inositol lost the ability (Fig. 4I). These results give a clear clue to explain how myo-inositol can depolarize the membrane potential of macrophages and why high doses of myo-inositol had an invert effect.

### Depolarized macrophages equally adhere to BLFX-S and BLFX-R

**Figure 5:**
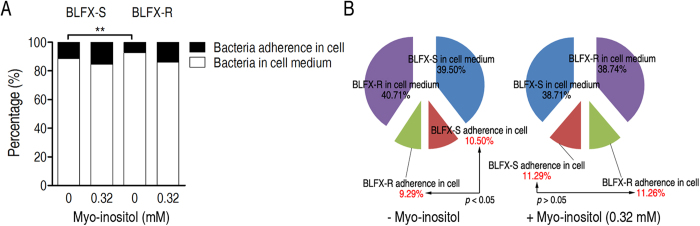
Myo-inositol enhances membrane potential-related adherence. (**A**) Percent adherence of BLFX-S or BLFX-R with RAW264.7 cells in the absence or presence of myo-inositol. (**B**) Percent adherence of a mixture of equal parts BLFX-S and BLFX-R with RAW264.7 cells in the presence or absence of myo-inositol. Error bars ± s .e .m, ^*^ *p* < 0.05, ^**^ *p* < 0.01.

We reasoned that macrophages depolarized by exogenous myo-inositol offset the lower polarized membrane potential of BLFX-R, thereby increasing the phagocytosis of BLFX-R (Fig. 3D). Given that membrane potential is directly related to cell adherence^23^, we used a macrophage adhesion assay to examine this hypothesis at 4 °C, a temperature that allows adherence but blocks phagocytosis. After separate incubations with BLFX-S and BLFX-R, myo-inositol significantly augmented the adherence of both strains to macrophages (Fig. 5A). When a mixture of equal doses of BLFX-R and BLFX-S was incubated with macrophages that were pretreated with or without myo-inositol, untreated macrophages exhibited an optimal attractive preference to BLFX-S, while myo-inositol-incubated macrophages showed an approximately equal attractive response to both bacteria (Fig. 5B). These results indicate that the myo-inositol-treated macrophages have an equal adherence to BLFX-S and BLFX-R, which is attributed to its depolarization.

### Model for myo-inositol equally promotes elimination of BLFX-S and BLFX-R

**Figure 6:**
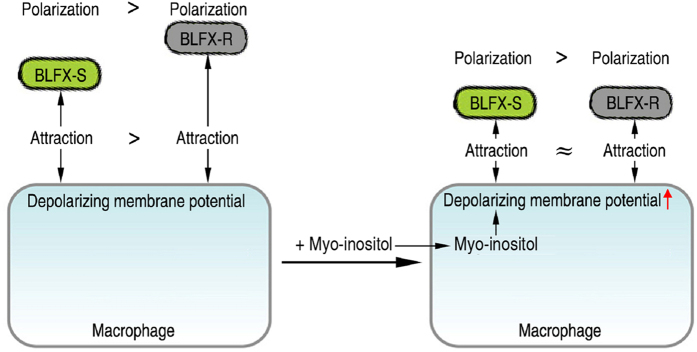
Model: Administration of myo-inositol overcomes host adaptation of BLFX-R. Myo-inositol enhances macrophage-mediated phagocytosis through depolarization of the macrophage membrane potential.

Although earlier reports revealed the inter-relationship between bacterial antibiotic resistance and membrane potential^24^,^25^, the membrane potential involving in host adaptation was previously unknown. Our current findings suggest that lower membrane polarization caused by antibiotic resistance promotes the host adaptation, thereby resulting in longer survival of BLFX-R in the host. More importantly, host is likely to overcome this host adaptation through increasing abundance of myo-inositol. A model of this overcoming strategy was summarized in Fig. 6. Briefly, due to the reduced polarization of BLFX-R compared to BLFX-S, macrophages have an optimal attractive preference to BLFX-S, which renders faster elimination of BLFX-S than BLFX-R. After administrating myo-inositol, macrophages are significantly depolarized. Besides elevating adhesion of BLFX-S and BLFX-R, depolarizing macrophages adhere to equal amount of BLFX-S and BLFX-R. In other words, the more depolarized BLFX-R is no longer the stubborn drug-resistant bacteria and can be eliminated easily like BLFX-S for depolarizing macrophages. Therefore, this model provides a feasible strategy to combat antibiotic-resistant bacteria through alteration of membrane potentials of macrophage.

## Methods

### Ethics statement

All work was conducted in strict accordance with the recommendations in the Guide for the Care and Use of Laboratory Animals of the National Institutes of Health. The protocol was approved by the Institutional Animal Care and Use Committee of Sun Yat-sen University (Animal Welfare Assurance Number: I6).

### Bacterial strains and culture conditions

The bacterial strains used in the present study consisted of *E. coli* K12 BW25113 and its derivative BLFX-sensitive strain (BLFX-S). The BLFX-resistant strain (BLFX-R) was selected by the use of serial propagations of BLFX-S in a Luria Bertani (LB) medium with a 1/2 minimum inhibitory concentration (MIC) of BLFX. The bacterial strain was cultured in LB medium at 37 °C. The minimum inhibitory concentration (MIC) was determined by antimicrobial susceptibility testing by allowing the bacteria to grow until it reached an optical density of 0.5 (OD600 nm), and then serial propagations were subcultured in LB medium.

### Zebrafish survival experiment

A zebrafish model was used to investigate the difference in pathogenicity between the BLFX-R and BLFX-S strains. Zebrafish were randomly divided into three groups with 20 fish in each group. These fish were challenged with BLFX-R, BLFX-S (both 5 × 10^7^ CFU/fish) or saline solution (control) and observed for 15 days to examine their survival.

### Bacterial elimination in a mouse model

Kunming mice weighing 22 ± 2 g were provided by the Center for Experimental Animals of Sun Yat-sen University, and animals were acclimated for one week. Bacterial cells from overnight cultures were diluted 1:100 into 100 mL of LB medium. The cultures were harvested at an absorbance of 1.0 (OD600) by centrifugation at 4000 x *g* for 15 min at 4 °C. The cells were washed in 40 mL of sterile saline (0.85% NaCl) and then resuspended in 0.85% NaCl. Each mouse was intraperitoneally infected by inoculation with 5 × 10^7^ CFU of bacteria. These mice were divided into two groups. One group was used to examine bacterial elimination, and the other group was used to investigate proteomics and metabolomics, which will be described later. For the bacterial elimination group, after bacterial injection, the mice were euthanized at 3 or 24 h after injection for collection of the spleen and liver. These organs were homogenized and the supernatant was used to examine bacterial cells via viable plate-counting. The maintenance and treatment of the experimental mice was approved by Sun Yat-sen University.

### Extraction of proteins and metabolites of the spleen

To investigate of the proteomics and metabolomics data, mice were randomly divided into two treatment groups and a control group. The two treatment groups were intraperitoneally infected by inoculation with 5 × 10^7^ CFU of BLFX-R or BLFX-S, and the control group was intraperitoneally injected with an equal volume of saline. The mice were euthanized after 3 h, and the spleens were harvested for proteomic and metabolomic investigation.

For the proteomic investigation, the spleen tissues were homogenized and dissolved for 4 h in lysis buffer (7 M urea, 2 M thiourea, 4% CHAPS, 60 mM DTT, 10 mM Tris, 1 mM EDTA, 0.5% ampholyte 3–10, and 1% protease inhibitor cocktail). Next, the homogenates were centrifuged at 9000 rpm for 1 h at 4 °C to remove debris, and the supernatants were collected for two-dimensional gel electrophoresis (2-DE) and stored at –80 °C until use. The concentration of protein in the final preparation was determined by the Bradford method^26^.

For the metabolomic investigation, the spleen tissue was homogenized and dissolved for 30 s in methanol at 4 °C. To extract metabolites, a volume of 500 μL of methanol was used for each 100 mg of spleen tissue in the sample. The homogenates were centrifuged at 12,000 x *g* for 10 min at 4 °C. The resulting supernatant (300 μL) was transferred to a GC sampling vial containing ribitol (10 μL, 0.1 mg/mL), and an internal standard and then dried in a vacuum centrifuge concentrator before the subsequent derivatization^27^.

### 2-DE and mass spectrometric (MS) analysis

2-DE was performed according to a procedure described previously^28^. Briefly, protein extracts of spleen containing 800 μg of proteins were dissolved in lysis solution (8 M urea, 2 M thiourea, 4% CHAPS, and 80 mM DTT). Isoelectric focusing (IEF, 0.02 cm × 17 cm) was performed with an IPGphor at 20 °C for 60 kVh. After IEF, IPG strips were equilibrated by two steps in buffer containing 7 M urea, 2% SDS, 50 mM Tris (pH 8.8) and 10% glycerol plus either 50 mM DTT for reduction or 100 mM iodoacetamide for alkylation. The equilibrated strips were subjected to two-dimensional electrophoresis using a 12% acrylamide gel. The preparative gels were stained with Coomassie brilliant blue G-250. Subsequently, gels were scanned with ImageScan (Amersham Biosciences, Uppsala, Sweden), and the raw images were analyzed using the 2-DE software ImageMaster version 5.0. Significantly altered spots at abundance were selected for mass spectrometric analysis, including those that showed a rate increase or decrease > 2-fold or complete appearance or disappearance. All MALDI analyses were performed with a fuzzy logic feedback control system (ReflexIII MALDI-TOF system, Bruker) equipped with delayed ion extraction. Peptide masses were compared against the NCBI database using Mascot ( http://www.matrixscience.com); the Mus musculus protein database was identified as a matching species, and the mass tolerance was 100 ppm. Protein scores greater than 64 were significant (*P* < 0.05).

### Derivatization and GC-MS analysis

Samples must be derived before GC-MS analysis; therefore, 80 μL of methoxamine/pyridine hydrochloride (20 mg/mL) was added to dried samples to induce oximation for 1.5 h at 37 °C, then 80 μL of the derivatization reagent MSTFA (Sigma) was mixed and reacted with the sample for 0.5 h at 37 °C. A 1-μL aliquot of the derivative of the supernatant was added to a tube and analyzed using GC-MS (Trace DSQ II, Thermo Scientific). The separation conditions of GC-MS consisted of an initial temperature of 70 °C (5 min) with a uniform increase to 270 °C at a speed of 2 °C /min (5 min); 0.5 μL sample volume, splitless injection; injection temperature, 270 °C; interface temperature, 270 °C; ion source (EI) temperature, 30 °C; ionization voltage, 70eV; quadrupole temperature, 150 °C; carrier gas, highly pure helium; velocity, 1.0 mL/min; and full scan way, 60–600 *m*/*z*.

### Statistical and bioinformatics analysis

Proteomic and metabolomic data were obtained using Melanie 4.0 and Thermo Foundation 1.0.1, respectively. The resulting data matrix was normalized using the sum abundance value, and then we centered the computed abundance of protein spots or metabolites for each tissue sample on their median value and scaled by their inter-quartile range (IQR) to reduce between-sample variation^29^,^30^. Prior to integrated analysis, we separately analyzed the differential proteins and metabolites from BLFX-S and BLFX-S using significant analysis of microarray (SAM), a permutation-based hypothesis testing method for the analysis of proteomic and metabolomic data^31^,^32^. Integrated analysis was performed as described previously^17^. The combined proteins and metabolites are displayed as saline-based *z*-score plots^29^. Independent component analysis (ICA) was selected as the pattern recognition method^17^,^33^.

### Measurement of bacterial elimination in the presence of exogenous myo-inositol

Mice weighing 22 ± 2 g were acclimatized for one week. Mice in both the test and control groups were intraperitoneally injected with 100 μL of myo-inositol or saline twice a day. Three days after injection, the mice were intraperitoneally injected with 5 × 10^7^ CFU of bacteria. Twelve hours later, the mice were euthanized by decapitation. The spleen and liver tissue were harvested and homogenized with sterile saline (0.85% NaCl). The homogenates were serially diluted in sterile saline, and the number of bacteria after 12 h of growth on solid media was counted. Differences between the groups were tested for significance (for small numbers) at two significance levels (0.05 and 0.01) using the statistical software SPSS (Statistical Package for the Social Sciences).

### Cell culture and quantitative phagocytosis assay

The murine macrophage cell line RAW264.7 was cultured at 37 °C in a 5% CO_2_ incubator in DMEM (Hyclone) supplemented with 10% (V/V) cosmic calf serum (Hyclone), 100 U/mL penicillin G and 100 U/mL streptomycin. Macrophage phagocytosis was examined as described previously^34^,^35^. Briefly, RAW264.7 cells were harvested using CaCl_2_- and MgCl_2_-free PBS with 5 mM EDTA and plated with 5 × 10^6^ macrophages/well in 6-well plates. Before phagocytosis, BLFX-S and BLFX-R were conjugated with FITC and treated with CCCP (20 μM). These two strains were centrifuged onto macrophages at a multiplicity of infection (MOI) of 100 in DMEM without serum or antibiotics. Then, plates were placed at 37 °C for 1.5 h. After incubation, the macrophages were vigorously washed with cold PBS to stop additional bacterial uptake or the destruction of bacteria in the phagosome. Cells were washed at least four times in cold PBS and then fixed in 4% paraformaldehyde before being harvested in cold PBS containing 5 mM EDTA and subjected to FACS^®^ analysis.

In experiments where CCCP or/and indicated concentrations of myo-inositol were added, the cells were deprived of serum overnight and then singly or additively incubated with the above-mentioned molecules in serum-starved medium (DMEM/0.5% serum). After pretreating for 6 h, the *E. coli*-GFP, FITC-conjugated BLFX-S strain or the FITC-conjugated BLFX-R strain were centrifuged onto macrophages at a multiplicity of infection (MOI) of 100 in DMEM without serum or antibiotics, and plates were placed at 37 °C for 1.5 h. After incubation, the macrophages were vigorously washed in cold PBS and fixed with 4% paraformaldehyde. Cells were analyzed using a BD BioSciences FACSCalibur flow cytometer using CellQuest software.

### Macrophage adherence assay

The macrophage adherence assay was based on the phagocytosis procedure with some modifications. Briefly, RAW264.7 cells were harvested and plated at 5 × 10^6^ macrophages/well in 6-well plates. For experiments in which myo-inositol was administered, the cells were deprived of serum overnight and then incubated with myo-inositol in serum-starved medium (DMEM/0.5% serum) for 6 h 37 °C. After pretreatment, FITC-conjugated BLFX-S, BLFX-R or a mixture of equal parts BLFX-R and BLFX-S were centrifuged onto macrophages at a multiplicity of infection (MOI) of 100 in DMEM without serum or antibiotics, and plates were placed at 4 °C for 1.5 h. After infection, macrophages were vigorously washed with 3 mL of cold PBS and harvested in 1 mL of ice-cold PBS. These two fractions of PBS solution were serially diluted in sterile saline, and bacteria were counted when a single colony appeared in the media after growth at 37 °C.

### Bacterial voltage staining

DiOC2(3), a fluorescent membrane-potential indicator dye, was applied to investigate the bacterial membrane potential. Bacteria were obtained from log-phase cultures and diluted to approximately 1 × 10^6^ cells per mL in filtered PBS. A volume of 10 μL of 3 mM DiOC2(3) was added to a 1 mL aliquot of the bacterial suspension, which was then incubated at room temperature for 30 min. Stained bacteria were assayed with a flow cytometer equipped with a laser emitting at 488 nm. Fluorescence was measured on the green and red channels (“GC” and “RC”), and filters were used to detect fluorescein and Texas Red dye, respectively. Because the relative amount of red and green fluorescence intensity varied with cell size and aggregation, the ratio of red to green fluorescence intensity was used as a size-independent indicator of membrane potential.

### Macrophage cell voltage staining

Intracellular voltage was examined using bis-(1,3-diethylthiobarbituric acid) trimethine oxonol (DiSBAC_2_(3) (Molecular Probes), a slow-response potential-sensitive probe. This probe enters depolarized cells and exhibits enhanced fluorescence. Enhanced depolarization leads to additional afflux of the anionic dye and an enhancement in fluorescence. In contrast, hyperpolarization is indicated by a reduction in fluorescence. Stock solutions of DiSBAC_2_(3) consisted of 0.5 mg/mL in a DMSO (Sigma). Cells were resuspended in citrate buffer (50 mM Na-citrate and 100 mM NaCl, pH 7.4) for collection, yielding approximately 10^7^ cells / mL. Then, 3 μL of stock solution was added to 1 mL of cell suspension, and samples were subsequently incubated for 20 min in darkness and subjected to FACS^®^ analysis. The ratio of fluorescence intensities excited at 530 nm was monitored at an emission wavelength of 560 nm.

## Additional Information

**How to cite this article**: Chen, X.-h. *et al.* Myo-inositol improves the host’s ability to eliminate balofloxacin-resistant *Escherichia coli*. *Sci. Rep.* **5**, 10720; doi: 10.1038/srep10720 (2015).

## Supplementary Material

Supplementary Information
