# Supplementary material for: Myo-inositol improves the host’s ability to eliminate balofloxacin-resistant Escherichia coli
Source: Sci Rep. 2015 Jun 1;5:10720. doi: 10.1038/srep10720 (PMC5377236; doi:10.1038/srep10720)
Supplement: Supplementary Information [file srep10720-s1.pdf]

Supplemental Information

Myo-inositol improves the host's ability to eliminate balofloxacin-resistant *Escherichia coli*

Xin-hai Chen , Bing-wen Zhang , Hui Li , Xuan-xian Peng

Supplementary Table Legends

Table S1. Identification of the differentially expressed protein spots on 2-DE gel using MALDI-TOF/MS

Spot number	NCBI accession no.	Protein description	Gene	MW(Da)/PI	MASCOT score	MASCOT Expect	Peptides matched	Cover %
1	gi|6755863	Endoplasmin (94 kDa glucose-regulated protein)	Hsp90b1	92703/4.74	147	2.90E-10	23	2
2	gi|40556608	Heat shock protein HSP 90-beta	Hsp90ab1	83571/4.97	218	2.30E-17	27	35
3	gi|148664759	IK cytokine	Ik	65950/6.26	101	1.10E-05	16	32
4	gi|254540166	78 kDa glucose-regulated protein	Hspa5	72492/5.07	262	9.10E-22	28	45
5	gi|254540166	78 kDa glucose-regulated protein	Hspa5	72377/5.07	837	2.90E-79	27	42
6	gi|254540166	78 kDa glucose-regulated protein	Hspa5	72377/5.07	372	9.10E-33	41	58
7	gi|42542422	Heat shock cognate 71 kDa protein	Hspa8	71056/5.28	117	2.90E-07	18	33
8	gi|124486724	Protein disulfide-isomerase A2	Pdia2	58280/4.87	288	2.30E-24	10	15
9	gi|31543113	plastin-2 [Mus musculus	Lcp1	70732/5.20	328	2.30E-28	41	63
10	gi|50815	Eukaryotic initiation factor 4A-I	Eif4a1	42386/6.56	182	9.10E-14	27	48
11	gi|9506571	Eukaryotic translation initiation factor 2 subunit 1	Eif2s1	36371/5.02	143	7.20E-10	20	47
12	gi|161086971	F-actin-capping protein subunit alpha-1	Capza1	33104/5.34	196	3.6E-15	17	80
13	gi|309090	A-X actin	Actb	42009/5.21	156	3.60E-11	14	43
14	gi|13385006	Cytochrome c1, heme protein, mitochondrial	Cyc1	35305/9.24	156	3.60E-11	3	16
15	gi|6678439	anionic trypsin-2 precursor	Prss2	26871/4.40	266	3.60E-22	3	20
16	gi|5453555	GTP-binding nuclear protein Ran	RAN	24579/7.01	188	2.30E-14	15	50
17	gi|255522937	Chymotrypsinogen B	Ctrb1	27804/4.91	527	2.90E-48	6	29
18	gi|255522937	Chymotrypsinogen B	Ctrb1	27804/4.91	360	1.40E-31	5	28
19	gi|51010909	trypsin 5 precursor	Try5	26944/5.06	299	1.80E-25	5	21

20	gi|6681079	cathepsin B preproprotein	Ctsb	38168/5.57	330	1.40E-28	9	28
21	gi|61402210	Apoa1 protein	Apoa1	23008/7.00	165	3.60E-12	8	39
22	gi|198278553	myosin regulatory light polypeptide 9	Myl9	19898/4.80	196	3.60E-15	3	18
23	gi|13994195	serine/threonine-protein phosphatase PP1-alpha catalytic subunit	Ppp1ca	38257/5.94	721	1.10E-67	24	60
24	gi|55715891	Vat1 protein, partial	Vat1	42723/5.96	150	1.40E-10	20	41
25	gi|111607467	Pancreatic alpha-amylase	Amy2	57966/6.88	249	1.80E-20	32	61
26	gi|6671539	Fructose-bisphosphate aldolase A	Aldoa	39787/8.31	162	9.10E-12	19	56
27	gi|38328278	Hnrpa3 protein	Hnrpa3	34584/9.08	400	1.40E-35	12	47
28	gi|6754976	Peroxiredoxin-1	Prdx1	22390/8.26	93	6.60E-05	4	25
29	gi|5031595	actin-related protein 2/3 complex subunit 4 isoform	ARPC4	19768/8.53	110	1.40E-06	10	66
30	gi|21465677	lysozyme C-2 precursor	Lyz2	17134/9.11	135	4.60E-09	11	78

Supplementary Figures and Legends

Figure S1

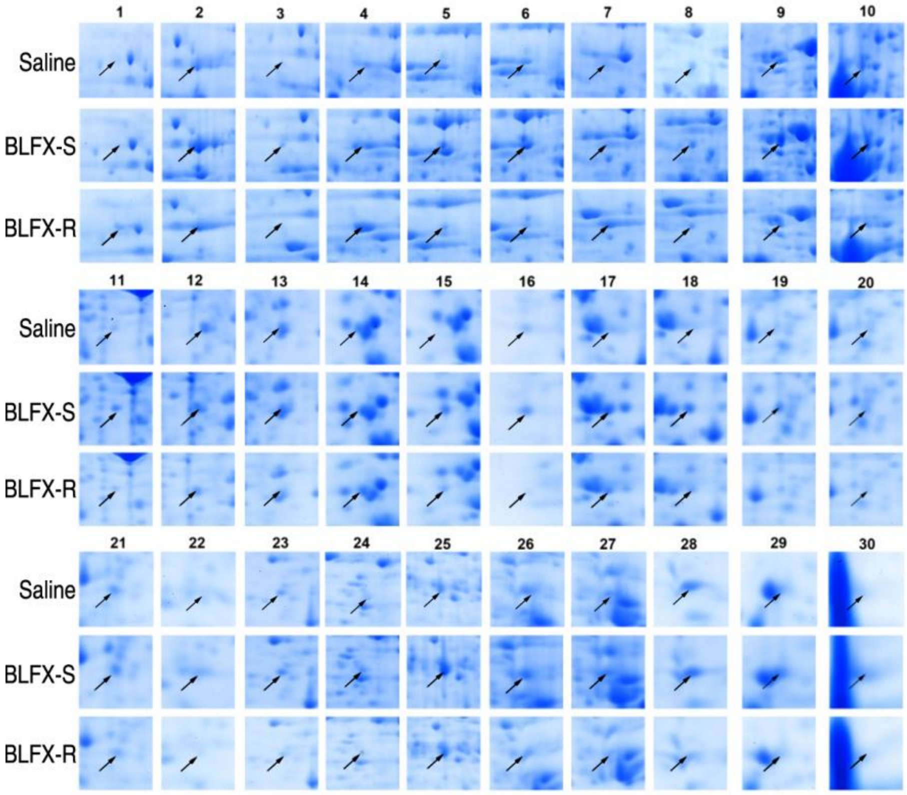

Figure S1. 2-DE based proteomics for investigation of differential proteins of spleen in response to BLFX-R infection. Enlarged partial 2-DE gels showing 30 differential expression spots.

Figure S2.

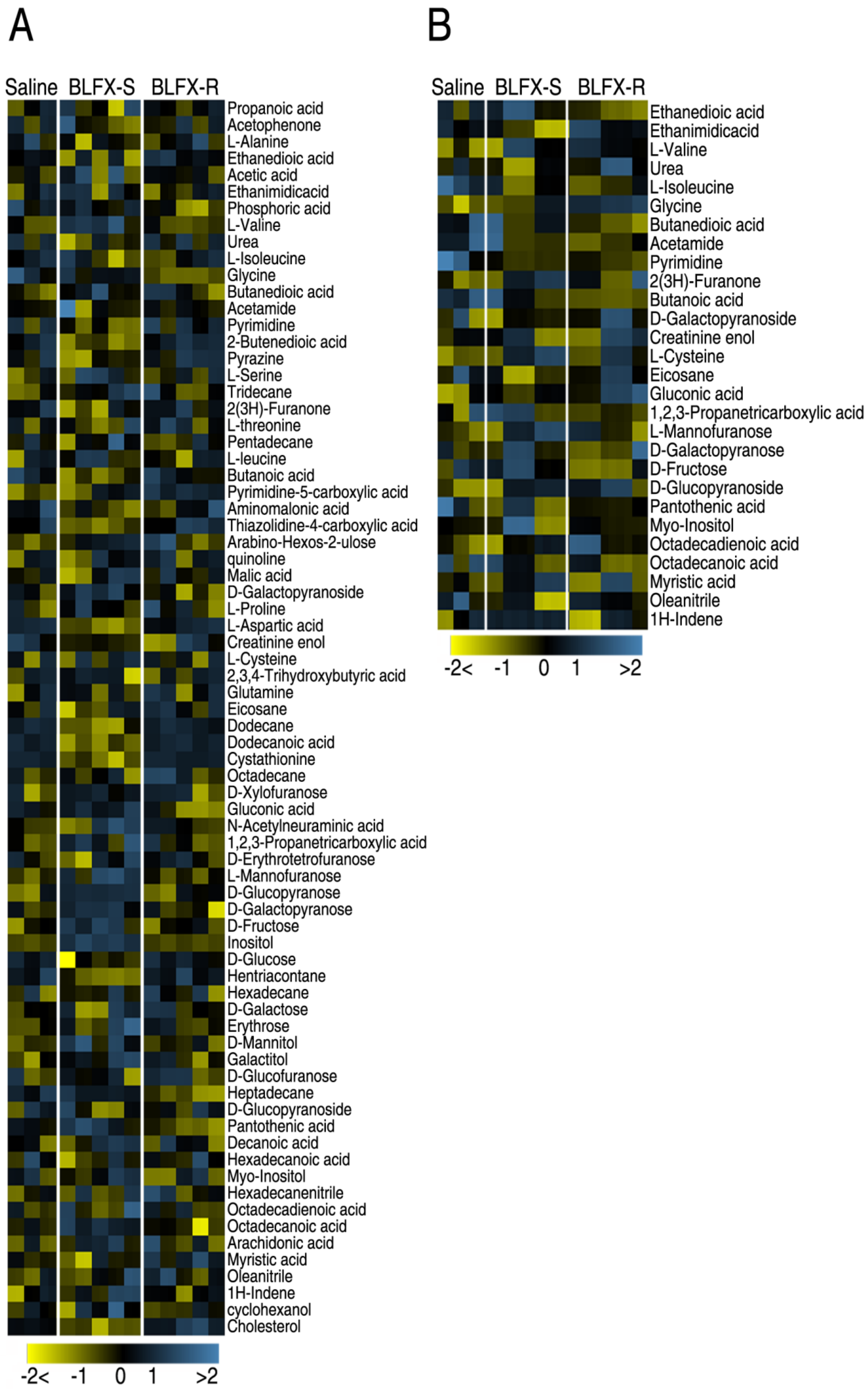

Figure S2. Differential abundance of metabolic profile in response to BLFX-R.

(A) Unsupervised hierarchical clustering analysis of 72 metabolites detected in spleen tissues. Dendrogram representing unsupervised hierarchical clustering of 13 spleen tissues described in Materials and Methods. Two technical replicates were prepared for each tissue. (B) Unsupervised hierarchical clustering analysis of 28 differential metabolites.
